# Neobavaisoflavone Induces Bilirubin Metabolizing Enzyme UGT1A1 via PPARα and PPARγ

**DOI:** 10.3389/fphar.2020.628314

**Published:** 2021-02-08

**Authors:** Ya-Di Zhu, Xiao-Qing Guan, Jing Chen, Sheng Peng, Moshe Finel, Ying-Yuan Zhao, Rui-Min Wang, Hui-Chang Bi, Ming Lei, Dan-Dan Wang, Guang-Bo Ge

**Affiliations:** ^1^ Trauma Emergency Center, The Seventh Affiliated People’s Hospital of Shanghai University of Traditional Chinese Medicine, Shanghai, China; ^2^ Institute of Interdisciplinary Integrative Medicine Research, Shanghai University of Traditional Chinese Medicine, Shanghai, China; ^3^ Division of Pharmaceutical Chemistry and Technology, Faculty of Pharmacy, University of Helsinki, Helsinki, Finland; ^4^ School of Pharmaceutical Sciences, Sun Yat-sen University, Guangzhou, China

**Keywords:** UDP-glucuronosyltransferase 1A1, flavonoids, induction, peroxisome proliferator-activated receptors, neobavaisoflavone

## Abstract

UDP-glucuronosyltransferase 1A1 (UGT1A1) is an essential enzyme in mammals that is responsible for detoxification and metabolic clearance of the endogenous toxin bilirubin and a variety of xenobiotics, including some crucial therapeutic drugs. Discovery of potent and safe UGT1A1 inducers will provide an alternative therapy for ameliorating hyperbilirubinaemia and drug-induced hepatoxicity. This study aims to find efficacious UGT1A1 inducer(s) from natural flavonoids, and to reveal the mechanism involved in up-regulating of this key conjugative enzyme by the flavonoid(s) with strong UGT1A1 induction activity. Among all the tested flavonoids, neobavaisoflavone (NBIF) displayed the most potent UGT1A1 induction activity, while its inductive effects were confirmed by both western blot and glucuronidation activity assays. A panel of nuclear receptor reporter assays demonstrated that NBIF activated PPARα and PPARγ in a dose-dependent manner. Meanwhile, we also found that NBIF could up-regulate the expression of PPARα and PPARγ in hepatic cells, suggesting that the induction of UGT1A1 by NBIF was mainly mediated by PPARs. In silico simulations showed that NBIF could stably bind on pocket II of PPARα and PPARγ. Collectively, our results demonstrated that NBIF is a natural inducer of UGT1A1, while this agent induced UGT1A1 mainly via activating and up-regulating PPARα and PPARγ. These findings suggested that NBIF can be used as a promising lead compound for the development of more efficacious UGT1A1 inducers to treat hyperbilirubinaemia and UGT1A1-associated drug toxicities.

## Introduction

Mammalian UDP-glucuronosyltransferase enzymes (UGTs) are a superfamily of xenobiotic-metabolizing enzymes that are located in the endoplasmic reticulum of epithelial cells in various tissues, particularly in the liver, small intestine and kidney (Fisher et al., 2001; Guillemette et al., 2014). UGTs catalyzed glucuronidation reaction of a wide range of compounds, such biotransformation increases the hydrophilicity of the substrates and facilitates their biliary and urinary excretion (Tukey and Strassburg, 2000a; Tukey and Strassburg, 2000b). UGT1A1, one of the most abundant and essential UGT enzymes in mammals, plays a crucial role in detoxification and metabolic clearance of a wide variety of xenobiotics and endogenous substances, such as bilirubin, bile acids and inflammatory mediators (Bosma 2003; Kiang et al., 2005; McDonagh, 2007; O’Dwyer and Catalano, 2006). Bilirubin, the final product of heme metabolism, is an endogenous toxin since failure to conjugate and dispose it may trigger hyperbilirubinaemia, liver injury and even irreversible brain damage at high concentrations (Roald et al., 2000; McDonagh, 2007). Increasing evidence has demonstrated that up-regulation of hepatic and intestinal UGT1A1 may accelerate the metabolic clearance of bilirubin or other UGT1A1-substrates, which in turn, provide alternative therapeutic therapy for the treatment of hyperbilirubinaemia and drug-induced liver toxicity (Galijatovic et al., 2001; Sticova and Jirsa, 2013; Goon et al., 2016). Therefore, it is highly desirable to find more efficacious UGT1A1 inducers as novel anti-hyperbilirubinaemia agents for the treatment of UGT1A1-associated human diseases and drug-induced liver toxicity (Aoshima et al., 2014; Gong et al., 2014; Zeng et al., 2016).

In view of the fact that natural compounds are one of the major sources for the discovery of lead compounds and drug candidates (RiceEvans et al., 1996; Middleton et al., 2000; Havsteen, 2002), it is more likely to find UGT1A1 inducers from natural products. In fact, a variety of natural compounds with diverse structures (such as acacetin, apigenin) have been reported with UGT1A1 inductive effects (Walle and Walle, 2002; Sugatani et al., 2004), which inspired us to discover more efficacious UGT1A1 inducers from natural compounds, especially those compounds with good safety profiles. From the viewpoints of drug discovery and translational applications, ideal UGT1A1 inducers should have the following features, such as high efficacy, low toxicity, cell-permeability (capable of targeting the nuclear receptors located in the cell cytoplasm) and acceptable metabolic stability. Unfortunately, most of the reported UGT1A1 inducers (such as chrysin, diosmetin, β-naphthoflavone) display moderate inductive potency, low cell permeability and poor metabolic stability (Walle et al., 2000; Walle and Walle, 2002; Viegas et al., 2012; Wang et al., 2014; Tsai et al., 2018). Hence, for the prevention and treatment of hyperbilirubinemia and other UGT1A1-associated human diseases, it was necessary as well as challenging to find some promising UGT1A1 inducers that possess good safety profiles and drug-likeness properties.

This study aimed at finding efficacious UGT1A1 inducers from natural flavonoids and their derivatives, as well as to explore the molecular mechanism of the newly identified flavonoid-type UGT1A1 inducer(s). For this purpose, a series of natural and semi-synthetic flavonoids were collected and their UGT1A1 inductive effects were routinely screened in living cells. After screening of 40 flavonoids, neobavaisoflavone (NBIF), a typical phenolic hydroxylated isoflavonoid isolated from the seeds of *Psoralea corylifolia* L, displayed the most potent UGT1A1 inductive activity in HepG2 cells. This finding encouraged us to further investigate the inductive effects of UGT1A1 by NBIF in various human cell lines and the molecular mechanism involved in up-regulating of this key conjugative enzyme.

## Materials and Experimental

### Chemicals and Reagents

NHPN (purity≥98%) and NHPNG were synthesized by the authors according to our previously described scheme (Lv et al., 2017). All the tested flavonoids (purity >98%) were purchased from Chengdu Preferred Biotech Co., Ltd. Each flavonoid was dissolved in DMSO to prepare the stock solution (20 mM) and stored at −80°C until use. Caco-2 cells, HepG2 cells, and HEK293 cells were obtained from the American Type Culture Collection (ATCC). DMEM Glucose medium and fetal bovine serum were purchased from Hyclone. RNAiso Plus reagent, RNA PCR kit, and SYBR® Premix Ex Taq™ II Kit were obtained from Takara. UGT1A1 and GAPDH primers listed in Supplementary Table S1 were synthesized by Takara. pGMLV-PPAR-Luc, pGMLV-AhR-Luc, GR-GAL4-PPARα-LBD and GR-GAL4-PPARγ-LBD were developed by Genomeditech (Shanghai, China). The siRNAs were synthesized by Genomeditech. Steady-Glo® Reagent (E2510) was purchased from Promega (Madison, WI, USA). Ultrapure water purified by Milli-Q® Integral Water Purification System (Millipore, USA) was used throughout, while LC grade Acetonitrile (ACN), dimethylsulfoxide (DMSO), and formic acid were ordered from Sigma-Aldrich (St. Louis, MO, USA).

### Cell Culture and Treatment

Caco-2 and HepG2 cells were grown in DMEM/High Glucose medium supplemented with 10% fetal bovine serum and 1% Penicillin-Streptomycin Solution. The culture condition is constant temperature of 37°C, a humidified atmosphere of 95% air and 5% CO_2_. Fresh media were replaced every two days. For induction assay, chrysin was used as a positive control (Galijatovic et al., 1999). All the tested flavonoids were stored in DMSO and diluted to the indicated concentrations by culture medium. The negative control group was treated with the vehicle DMSO alone. The final concentration of DMSO in all experiments was below 1% (V/V).

### Real-Time PCR Assays

For related gene expression analysis, the HepG2 cells were seeded in 24-well plates at a density of 3×10^4^ cells/well. When cells reached about 50% confluence, chrysin or the tested flavonoids listed in Tables 1, 2 were added to culture medium and cultured for 72 h. All flavonoids were used at a final concentration of 25 μM, the optimum concentration for UGT1A1 induction by chrysin in HepG2 cells (Galijatovic et al., 2000). As for time- and dose-dependent UGT1A1 induction assays by neobavaisoflavone, the cells are divided into two groups. One group of cells were used to investigate the dose-dependent induction, namely the cells were treated with gradual incremental concentrations of NBIF (a final concentration of either 2 μM, 10 μM or 25 μM), respectively. Meanwhile, another group of cells were used to investigate the time-dependent induction, in which the cells were incubated with NBIF (25 μM, final concentration) for 0 h, 24 h, 48 h, and 72 h, respectively.

**TABLE 1:** The inductive effects of tested flavonoids on UGT1A1 gene expression. HepG2 cells were treated with each tested compound (25 µM) as stated above and the total RNA was isolated from cultures treated for three consecutive days. Transcripts of UGT1A1 genes were quantified by real-time PCR and normalized to GAPDH. Data represent the mean ± SEM. *p < 0.05 (student’s t-test) compared to control group.
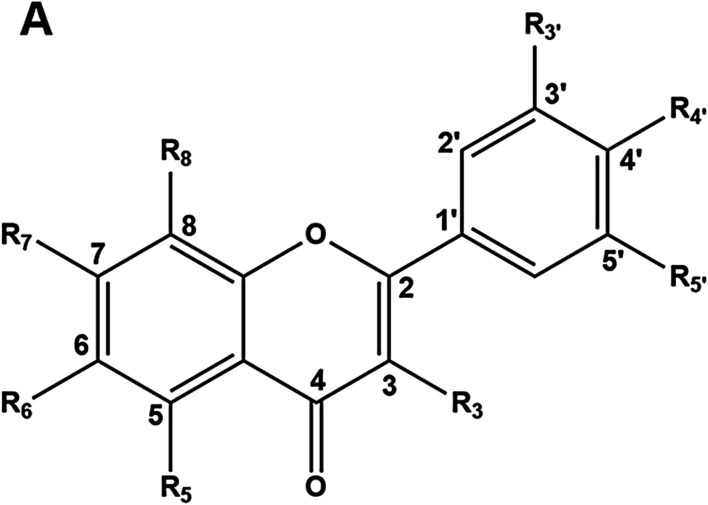

No	Compound	Skeleton	R_3_	R_5_	R_6_	R_7_	R_8_	R_3’_	R_4’_	R_5’_	n-folds of ctr
1	Chrysin	A	H	OH	H	OH	H	H	H	H	9.68 ± 0.31
2	3,6-Dihydroxyflavone	A	OH	H	OH	H	H	H	H	H	<0.01
3	3-Hydroxyflavone	A	OH	H	H	H	H	H	H	H	1.02 ± 0.03
4	6,7-Dimethoxybaicalein	A	H	OH	OCH_3_	OCH_3_	H	H	H	H	0.73 ± 0.01
5	Luteolin	A	H	OH	H	OH	H	OH	OH	H	0.74 ± 0.14
6	Apigenin	A	H	OH	H	OH	H	H	OH	H	0.22 ± 0.01
7	Apigenin 7-O-methyl ether	A	H	OH	H	OCH_3_	H	H	OH	H	0.95 ± 0.01
8	Hispidulin	A	H	OH	OCH_3_	OH	H	H	OH	H	0.91 ± 0.01
9	5,6-Dihydroxy-7-methoxyflavone	A	H	OH	OH	OCH_3_	H	H	H	H	0.90 ± 0.03
10	Norwogonin	A	H	OH	H	OH	OH	H	H	H	0.11 ± 0.01
11	Isovitexin	A	H	OH	Glu	H	H	H	OH	H	<0.01
12	Vitexin	A	H	OH	H	OH	Glu	H	OH	H	0.12 ± 0.01
13	Nobiletin	A	H	OCH_3_	OCH_3_	OCH_3_	OCH_3_	H	OCH_3_	OCH_3_	0.60 ± 0.08
14	Galangin	A	OH	OH	H	OH	H	H	H	H	0.20 ± 0.01
15	5,6,7-Trimethoxyflavone	A	H	OCH_3_	OCH_3_	OCH_3_	H	H	H	H	1.03 ± 0.06
16	6-Hydroxyflavone	A	H	H	OH	H	H	H	H	H	7.68 ± 0.27
17	7,8-Dihydroxyflavone	A	H	H	H	OH	OH	H	H	H	1.22 ± 0.13
18	Kaempferol	A	OH	OH	H	OH	H	H	OH	H	1.97 ± 0.10
19	Scutellarein	A	H	OH	OH	OH	H	H	OH	H	5.55 ± 0.04
20	Dihydromyricetin	A	OH	OH	H	OH	H	OH	OH	OH	3.22 ± 0.07
21	Hesperitin	A	H	OH	H	OH	H	H	OCH_3_	OH	7.02 ± 0.21
22	Fisetin	A	OH	H	H	OH	H	H	OH	OH	4.45 ± 0.15
23	Naringenin	A	H	OH	H	OH	H	H	OH	H	1.35 ± 0.09
24	4-Hydroxyflavone	A	H	OH	H	H	H	H	H	H	1.98 ± 0.18
25	5-Hydroxy-6-methoxyfalvone	A	H	OH	OCH_3_	H	H	H	H	H	0.81 ± 0.08
26	3′,4′-Dihydroxyflavone	A	H	H	H	H	H	OH	OH	H	0.43 ± 0.02
27	Wogonin	A	H	OH	H	OH	OCH_3_	H	H	H	1.67 ± 0.34
28	Oroxlin A	A	H	OH	OH	Glu	H	H	H	H	2.44 ± 0.09
29	Acacetin	A	H	OH	H	OH	H	H	OCH_3_	H	4.95 ± 0.08
30	Herbacetin	A	OH	OH	H	Glu	OH	H	OH	H	0.80 ± 0.01

**TABLE 2:** The inductive effects of tested isoflavonoids on UGT1A1 gene expression. HepG2 cells were treated with each tested compound (25 µM) as stated above and the total RNA was isolated from cultures treated for three consecutive days. Transcripts of UGT1A1 genes were quantified by real-time PCR and normalized to GAPDH. Data represent the mean ± SEM. *p < 0.05 (student’s t-test) compared to control group.
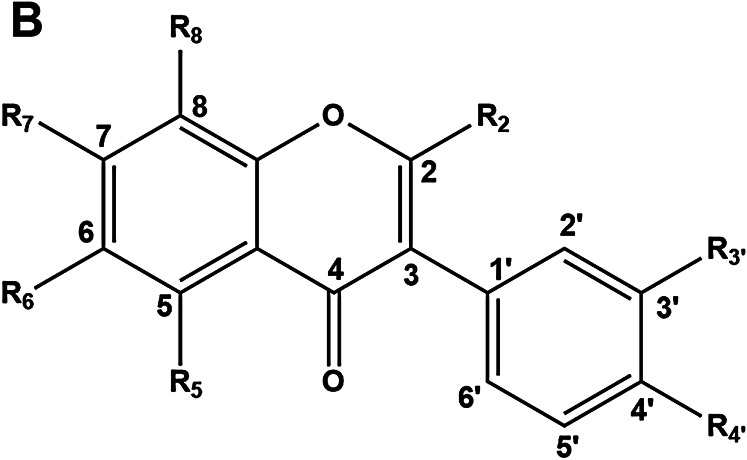

No	Compound	Skeleton	R_2_	R_5_	R_6_	R_7_	R_8_	R_3’_	R_4’_	n-folds of ctr
1	Isoflavone	B	H	H	H	H	H	H	H	1.11 ± 0.02
2	4',7-Dimethoxyisoflavone	B	H	H	H	OCH_3_	H	H	OCH_3_	2.67 ± 0.38
3	Ipriflavone	B	H	H	H	Isopropyloxy	H	H	H	1.53 ± 0.12
4	Corylifol A	B	H	H	H	OH	H	Geranyl	OH	1.51 ± 0.02
5	Calycosin	B	H	H	H	H	H	OH	OCH_3_	3.17 ± 0.05
6	Neobavaisoflavone	B	H	H	H	OH	H	Isopentenyl	OH	12.96 ± 0.18
7	Pueraria glycoside	B	H	H	H	OH	Glu	OH	OH	0.99 ± 0.13
8	Daidzin	B	H	H	H	Glu	H	H	OH	1.53 ± 0.02
9	Calycosin 7-*O*-glucoside	B	H	H	H	Glu	H	OH	OCH_3_	1.66 ± 0.06
10	Glycitin	B	H	H	OCH_3_	Glu	H	H	OH	1.62 ± 0.05

Before real-time PCR assays, HepG2 cells and Caco-2 cells were harvested with RNAiso Plus reagent and the total RNA was extracted as previously described (Li et al., 2013). The cDNA was synthesized from total RNA using RNA PCR kit following the supplier’s instructions. Then, the real-time PCR experiment was performed on ABI 7500 real-time PCR System utilizing SYBR® *Premix Ex Taq*™ II Kit according to the manufacturer’s instruction. Relative expression level for target gene was normalized by the Ct value of human GAPDH (2^△Ct^ formula). Values shown represent normalized relative fold changes of mRNA levels. Each sample was assayed in triplicate.

### Western Blot Assays

For UGT1A1 protein expression by western blot assay, Caco-2 cells and HepG2 cells were seeded into 6-well plates at a density of 1.2×10^6^ cells/well. Cell homogenates were prepared as described in our previous work (Lv et al., 2016). Before western blot assay, protein concentration was determined by the bicinchoninic acid assay (BCA) protein assay kit (Thermo Scientific, Rockford). Then cell homogenates were separated by 10% sodium dodecyl sulfate polyacrylamide gel electrophoresis. After that proteins were then transferred to PVDF membrane. The PVDF membrane was blocked with 5% nonfat dry milk in Tris-buffered saline, and further incubated with primary antibodies, either anti-UGT1A1 (ab194697, Abcam, dilution 1:1,000) or anti-GAPDH (G8795, Sigma Aldrich, dilution 1:1,000). Following overnight incubation with the primary antibodies, the membranes were washed with TBST buffer and incubated with the secondary antibodies (ab214879, ab214880, Abcam, dilution 1:1,000) for 1 h at room temperature and then washed in TBST again. Detection was conducted by GE Amersham Imager 600 (BioRad) with Meilunbio® fg super sensitive ECL luminescence reagent. The PPARα and PPARγ protein levels in the cell lysates were assayed by SimpleWestern blotting system according to a previously described scheme (Zhu et al., 2020a). Relative protein expression was then evaluated by measuring the optical density (OD) using Image Lab.

### UDP-Glucuronosyltransferase 1A1 Activity Assays in Living Cells

The UGT1A1 activity was determined *via* a probe reaction (NHPN *O*-glucuronidation), the process of which was modified slightly to determine UGT1A1 activity in intact HepG2 and Caco-2 cell culture systems (Lv et al., 2017). In short, the cell medium was discarded and the cultured cells were incubated with 50 µM NHPN after the final day of drug treatment. After 3 h incubation, 50 μL of culture medium from each well were taken and mixed with equal volume of ice-cold ACN. Then the mixtures were centrifuged at 20,000 g for 20 min at 4°C. The supernatant fraction was then subjected to Shimadzu LC-30 A liquid system to determine the production of NHPNG. The detective method was developed according to our previously described (Zhu et al., 2020b).

### Construction of Stable Transfection Cells

#### HEK293-PPARs -Luc and AhR -Luc Cell Lines

HEK293-PPARs-luc and HEK293-AhR-luc cell lines were developed *via* lentiviral stable transfection of pGMLV-PPARs-luc and pGMLV-AhR-luc, respectively. pGMLV-PPARs-luc and pGMLV-AhR-luc are kinds of plasmids that designed by Genomeditech, which harbors PPARs and AhR promoter-luciferase reporter gene construct driven by multiple dioxin response elements, respectively. After that, HEK293-PPARs-luc and AhR-luc cell lines were obtained through antibiotic screening.

#### HEK -293-PPARα-luc and PPARγ-luc Cell Lines

HEK293-PPARα-luc and HEK293-PPARγ-luc cell lines were constructed as isoform-specific luciferase reporter gene systems *via* lentiviral stable transfection of GR-GAL4-PPARα-LBD and GR-GAL4-PPARγ-LBD, respectively. GR-GAL4-PPARα-LBD and GR-GAL4-PPARγ-LBD are kinds of plasmids that designed by Genomeditech, which harbors PPARα and PPARγ promoter-luciferase reporter gene construct driven by multiple dioxin response elements, respectively. After that, HEK293-PPARα-luc and HEK293-PPARγ-luc cell lines cell lines were obtained through lentiviral stable transfection of GAL4-TATA-Luc-PGK-Puro and then the antibiotic screening were carried out.

### Reporter Gene Assays

#### PPARs and AhR Reporter Assays

For PPARs and AhR reporter gene activation detection, cells were split and grown according to the vendor’s instructions and plated into 96-well plates with incubation for 24 h. After discard the media, NBIF was added to a final concentration of either 2 μM, 10 μM or 25 μM, while vehicle control wells (0.1% DMSO) were used as negative control. Following 24 h incubation in the presence of the inducers, the media were discarded and the cells were washed with PBS. D-luciferin was then added to each well at a final concentration of 150 μg/ml. After incubation with D-luciferin at 37°C for 10 min, luciferase activity was measured in each well using Spectramax M3 (Molecular devices, USA). Rosiglitazone and luteolin have been reported as a strong agonist of PPARs (D’Aniello et al., 2019) and AhR (Koper et al., 2019), repectively, which were used as positive control in the present experiments.

#### PXR and FXR Reporter Assays

The PXR and FXR reporter gene activation assay were conducted based on our previously described method (Chen et al., 2014; Zeng et al., 2017). HEK293 cells were first seeded in 96-well plates at a density of 1.5 × 10^4^ cells per well without antibiotics treatment. For PXR reporter assays, each well were added 50 ng pSG5-PXR and 5 ng pRL-TK. While for FXR, each well contained 100 ng of tk-EcRE-Luc, 50 ng of FXR expression vector, and 5 ng of pRL-TK. The transfection procedure was followed the supplier’s instructions of Lipofectamine (Invitrogen, Grand Island, NY). Six hours after transfection, the transfection media were discarded and replaced with phenol red free-DMEM, which contained 10% charcoal-stripped delipidated FBS. Transfected cells were then treated with NBIF (a final concentration of either 2 μM, 10 μM or 25 μM) for 24 h. Rifampicin and chenodeoxycholic acid have been reported as a strong agonist of PXR and FXR, repectively, which were used as positive control in the present experiments. Luciferase activity was assayed in an Amersham Pharmacia Biotech luminometer using the Dual Reporter Assay System (Promega, Madison, WI) according to the manufacturer’s instructions. Renilla activity was employed as control and relative activation activity level for target nuclear receptor was normalized by the renilla luciferase activity.

#### PPARα and PPARγ Reporter Assays

To assign which PPAR isoform(s) was involved in UGT1A1 induction by NBIF, cells were plated into 96-well plates and NBIF was added to a final concentration of either 2 μM, 10 μM or 25 μM, while vehicle control wells (0.1% DMSO) were used as negative control. Following 24 h incubation, luciferase activity was measured by the Steady-Glo® Reagent (Promega) according to the manufacturer’s directions. Luciferase activity was measured on Spectramax M3 (Molecular devices, USA) with luciferase production as readout. Data was shown as the percentage of fold induction of the test compound against control group.

### Knocking Down PPARα and PPARγ by siRNA in Living HepG2 Cells

HepG2 cells growing in 24-well and 96-well plate were transiently transfected with siRNA targeting specific deadenylase subunits (siPPARα or siPPARγ, 50 nmol) by using Lipofectamine 3,000 (Thermo Fisher) according to manufacturer's protocol. And a nonspecific siRNA-treated group (siNC, 50 nmol) was used as negative control. After 24 h transfection, the cells were treated with NBIF (25 μM, final concentration) for three consecutive days. After the final day of induction treatment, the mRNA, protein expression and activity of UGT1A1 was examined as described above. The siRNAs sequences are provided in Supplementary Table S1.

### Molecular Docking and Molecular Dynamics Simulations

The ligand-binding domain (LBD) of PPARα (PDB ID: 2P54) and PPARγ (PDB ID:2F4B) (D’Aniello et al., 2019; Pettersson et al., 2007) were subjected to docking simulations using Discovery Studio (BIOVIA Discovery Studio 2016; Dassault Systèmes, San Diego, USA). The protein-ligand complexes with the highest LibDock score were taken as the initial structures for molecular dynamics simulation. Gromacs version 2016.5 was used to perform all the molecular dynamics simulations (Willson et al., 2000). The CHARMM36 all-atom force field parameters were used for proteins (Vanommeslaeghe et al., 2010), and the force field parameters of NBIF were generated by the CHARMM General Force Field (CGenFF) (Vanommeslaeghe and MacKerell, 2012). The force field and simulation parameters were conducted according to a previous publication (Guan et al., 2019). Trajectory analyses and visualization of structures were performed by the GROMACS package, PyMOL (Version 2.3, Schrödinger, LLC, New York City, USA) and Discovery Studio Visualizer (BIOVIA Discovery Studio 2019; Dassault Systèmes, SanDiego, USA). The total deviations of ligand atoms were analyzed by Root-Mean-Square Deviation (RMSD) by aligning to their initial docking structures after least square fit of proteins. The binding free energy was calculated by g_mmpbsa tool using the molecular mechanics Poisson Boltzmann surface area (MM-PB/SA) method (Kollman et al., 2000; Kumari et al., 2014). The entropy contributions were ignored due to relatively small structural fluctuations.

### Statistical Analysis

All data were expressed as mean ± standard deviation (SD) from triplicate assays. Statistical differences were determined by one-way ANOVA and differences were considered statistically significant at *p* value <0.05.

## Results

### Screening of UDP-Glucuronosyltransferase 1A1 Inductive Effects by Flavonoids at mRNA Levels

Firstly, the inductive effects on the mRNA levels of UGT1A1 of a panel of flavones and isoflavones (25 μM, final concentration) were assayed in living HepG2 cells. The results are shown separately for the tested flavones and isoflavones in Tables 1, 2, respectively. As shown in Table 1, four flavones exhibited relatively strong UGT1A1 inductive effects, including chrysin, 6-hydroxyflavone, hesperitin and scutellarein, inductive effects of 5-fold or higher than the negative control (DMSO only). Among all tested isoflavones, only neobavaisoflavone (NBIF) displayed strong UGT1A1 inductive effect, which induced nearly 13-fold enhancement on UGT1A1 expression at mRNA level when compared with the negative control (DMSO only). It should be noted that chrysin has been characterized in the past as a potent UGT1A1 inducer (Galijatovic et al., 2001), but our findings suggest that NBIF is a more potent UGT1A1 inducer (Tables 1, 2), at least in HepG2 cells. These results encouraged us to further investigate the UGT1A1 inductive effects and inductive mechanisms of this isopentenyl substituted isoflavone.

### Time- and Dose-Dependent UDP-Glucuronosyltransferase 1A1 Induction Assays by Neobavaisoflavone

**FIGURE 1:**
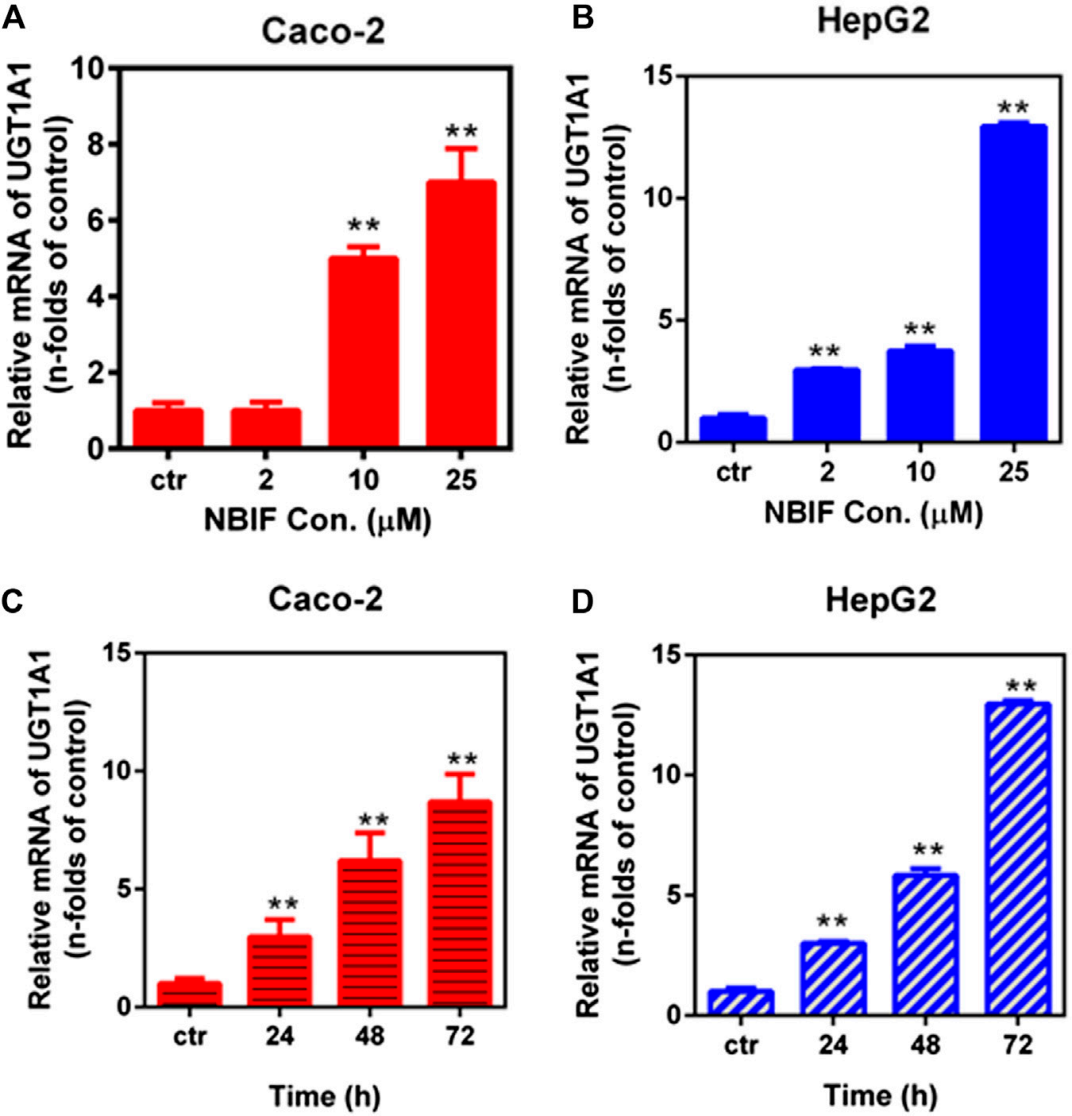
Induction of UGT1A1 by NBIF in both Caco-2 and HepG2 cell lines at the transcriptional level. **(A)** Dose-dependent activation of UGT1A1 mRNA expression by NBIF. Total RNA was extracted from the cells after three consecutive days. **(B)** Time-dependent activation of UGT1A1 mRNA expression in Caco-2 and HepG2 cells by NBIF (25 µM). Total RNA was extracted from the cells at each indicated time-point. *p < 0.05, **p < 0.01, significantly different from the negative control using one-way ANOVA analysis.

The time- and dose-dependent inductive effects of NBIF on UGT1A1 in both Caco-2 and HepG2 cells were subsequently investigated. As depicted in Figures 1A,B, NBIF dose-dependently induced UGT1A1 mRNA levels in both Caco-2 and HepG2 cells. HepG2 cells appeared to be more responsive to this agent, particularly at low concentration, but the induction was clear in both cell lines. The time-dependence of UGT1A1 mRNA induction levels by NBIF was time-dependent and nearly identical among these two cell lines **(** Figures 1C,D). Following incubation with NBIF for 72 h, NBIF could up-regulate the UGT1A1 mRNA levels in Caco-2 cells and HepG2 cells by 8.70 ± 0.31-fold, and 12.95 ± 0.18-fold, respectively. It appears from Figure 1 that NBIF induces UGT1A1 in a more efficient way in HepG2 cells when compared with Caco-2 cells, implying that NBIF might be more effective for modulating UGT1A1 in the human liver.

### Inductive Effects of Neobavaisoflavone on UDP-Glucuronosyltransferase 1A1 Protein and Activity Levels

**FIGURE 2:**
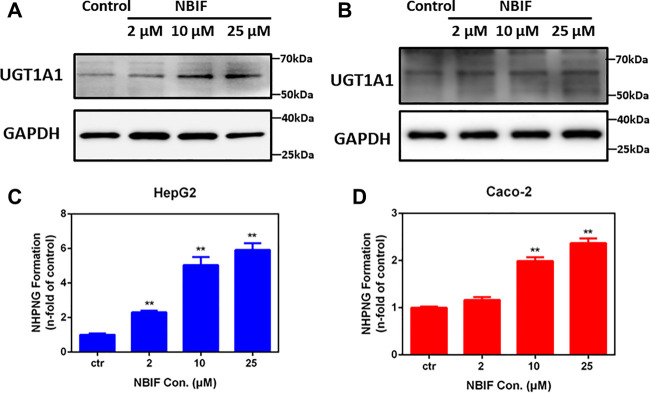
Inductive effects of NBIF on UGT1A1 expression. Western blotting of UGT1A1 in cell lysate from HepG2 **(A)** or Caco-2 **(B)** cells following treatment with DMSO (negative control) or NBIF (2, 10, 25 µM) for three consecutive days. UGT1A1 activity in HepG2 **(C)** and Caco-2**(D)** cells following treatment with NBIF (25 µM) for three consecutive days. **p < 0.01, significantly different from the negative control using one-way ANOVA analysis.

The inductive effects of NBIF were subsequently examined at the UGT1A1 protein level, in both Caco-2 and HepG2 cells. Following treatment with different concentrations of NBIF, all for 72 h, the level of expressed enzymes was determined using western blotting analysis (Figure 2). The UGT1A1 protein level was markedly increased in HepG2 cells following treatment with NBIF at a final concentration of 25 μM (Figure 2A). By contrast, the expression induction of UGT1A1 by NBIF at the protein level in Caco-2 cells was rather low, about 1.5-fold at 25 μM (Figure 2B).

UGT1A1 activity assay in these cells was another, independent way to examine the dose-dependent NBIF treatment on UGT1A1 expression. In this study, UGT1A1 activity were assayed using a fluorescence-based biochemical assay (Lv et al., 2017) and the results were shown in Figure 2C. Following treatment with NBIF for 72 h, UGT1A1 mediated NHPN-O-glucuronidation activity was significantly increased in HepG2 cells (about 6.0-fold at 25 μM) in comparison to the negative control (DMSO only). In Caco-2 cells, the UGT1A1 following treatment by the same NBIF concentration was increased by about 2.4-fold (Figure 2D). These findings are consistent with the results of UGT1A1 induction in both mRNA and protein levels, suggesting that NBIF up-regulated UGT1A1 in a more efficient way in HepG2 cells, particularly when it comes to active protein expression level.

### Identification of Nuclear Receptor(s) Involved in UDP-Glucuronosyltransferase 1A1 Induction by NBIF

**FIGURE 3:**
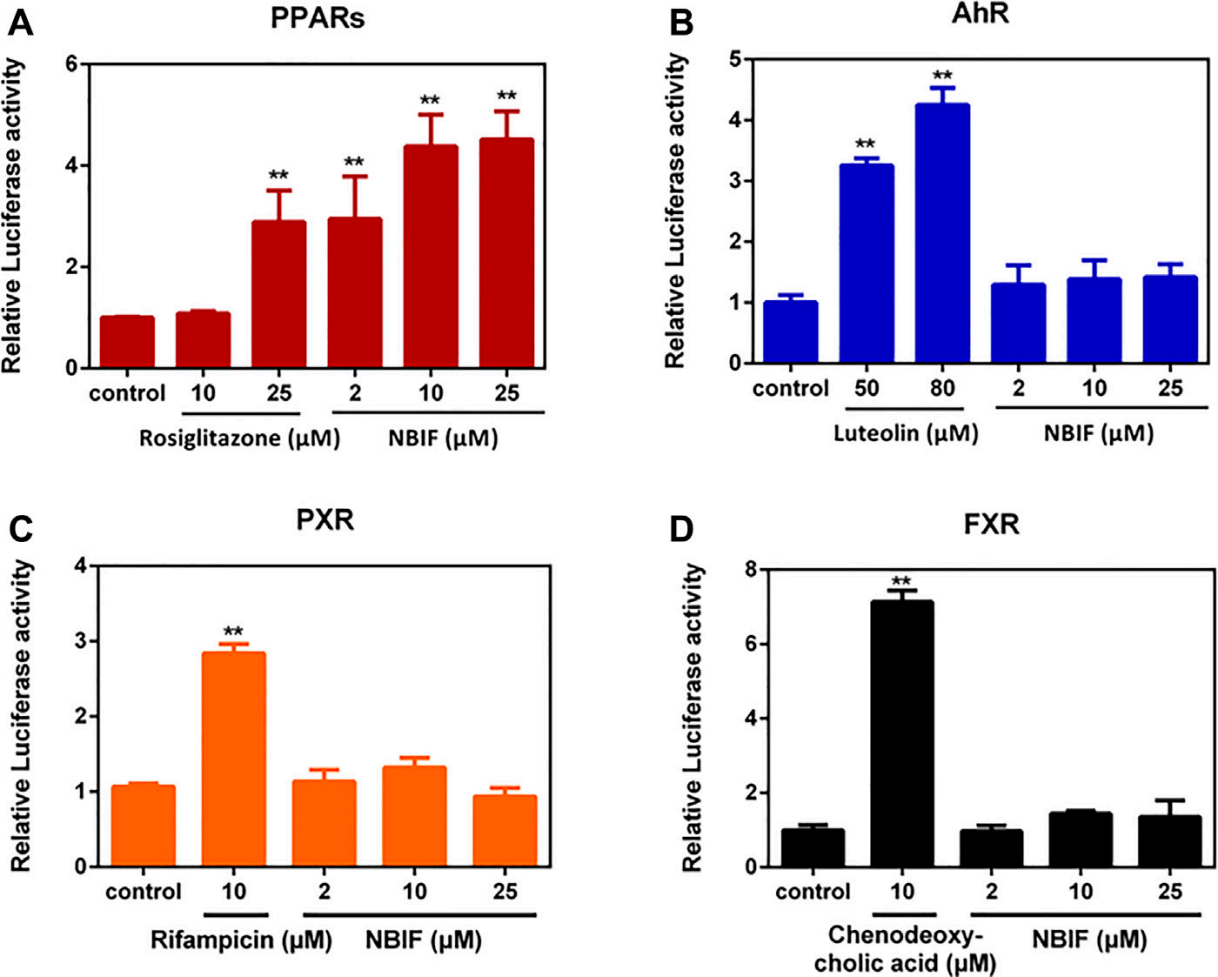
PPARs, AhR, PXR and FXR reporter gene activation with NBIF for 72 h. Rosiglitazone, luteolin, rifampicin, and chenodeoxycholic acid were used as the positive agonist of PPAR, AhR, PXR, and FXR, respectively. *p < 0.05, **p < 0.01, significantly different from the negative control using one-way ANOVA analysis.

It has been reported that the transcription of human UGT1A1 gene is mainly regulated by a panel of nuclear receptors (NRs), such as pregnane X receptor (PXR), farnesoid X receptor (FXR), peroxisome proliferator-activated receptors (PPARs) and aryl hydrocarbon receptor (AhR) (Sugatani et al., 2005; Kawase et al., 2007; Kohle and Bock, 2007; Chang et al., 2008; Yao et al., 2019). Thus, we examined whether NBIF could regulate promoter activity *via* activation of PPARs, PXR, FXR or AhR. As shown in Figure 3, NBIF significantly enhanced the luciferase activity of the PPAR reporter gene by about 4.5-fold at 25 μM, while the drug rosiglitazone, a known agonist of the human PPARs, enhanced PPARs reporter gene activity by about 2.9-fold at its highest dose 25 μM, such effect was quite similar to the stimulatory effect of 2 μM NBIF (Figure 3A). On the other hand, NBIF did not exhibit any significant activation of PXR, FXR or AhR reporter genes at the indicated concentrations, while the respective positive agonists obviously elevated the corresponding reporter activities (Figures 3B–D). These results suggest that NBIF up-regulates the transcription of human UGT1A1 *via* activation of PPARs rather than PXR, FXR or AhR.

### Assignment of the PPAR Isoform(s) Involved in UDP-Glucuronosyltransferase 1A1 Induction by Neobavaisoflavone

**FIGURE 4:**
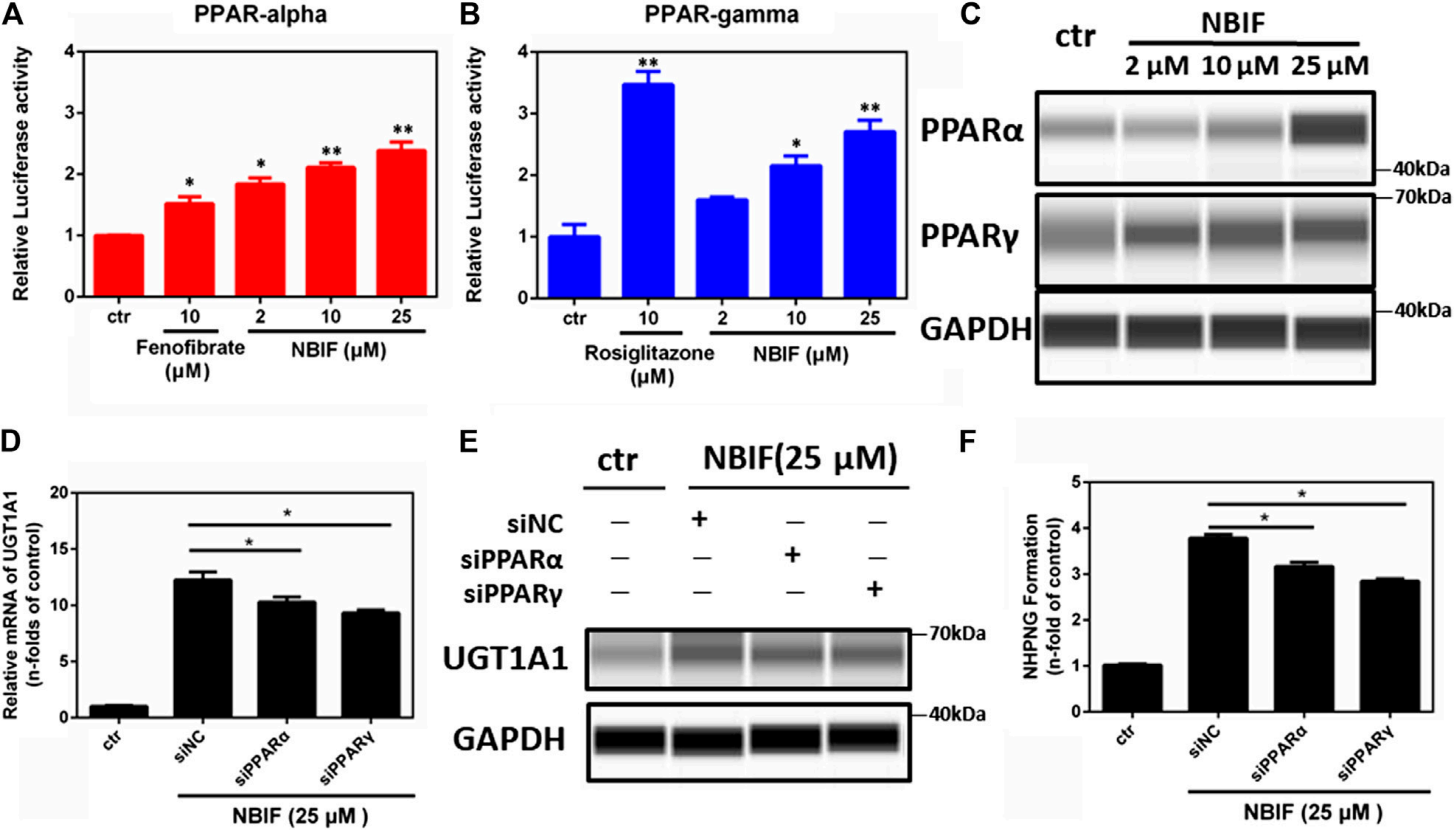
Effect of NBIF on activity and protein expressions of PPARα and PPARγ. **(A)** The agonist-like activities of NBIF on PPARα. **(B)** The agonist-like activities of NBIF on PPARγ. **(C)** Levels of PPARα and PPARγ protein in HepG2 cells treated with NBIF for three consecutive days were determined by Simple Wes. *p < 0.05, **p < 0.01, significantly different from the negative control using one-way ANOVA analysis.

underIt is well-known that three PPAR isoform(s) have been reported in the human body, while PPARα and PPARγ were recognized as the key target for inducing a variety of metabolic enzymes in mammals (Elshazly and Soliman, 2019). In order to explore whether PPARα or PPARγ could be activated by NBIF, the activation effects of PPARα and PPARγ was subsequently examined using luciferase reporter assay. Does-dependent assays for the effect of NBIF on the expression of each of PPARα and PPARγ was carried out, using suitable positive control for each one, in addition to the negative control. The results reveal that NBIF significantly enhance the reporter activities in cells transfected with PPARα, and PPARγ (Figure 4). In comparison with the vehicle group, NBIF (25 μM) resulted in the enhancement of luciferase activity of PPARα, and PPARγ reporters for 2.35 ± 0.11 and 2.72 ± 0.07-fold, respectively. These results suggest that NBIF is a new PPARα/γ dual agonists, which can up-regulate the transcription of human UGT1A1 via activating of PPARα and PPARγ.

### Neobavaisoflavone Up-Regulates PPARα and PPARγ Protein Expression

Furthermore, we also investigated whether NBIF could induce the protein expression of PPARα and PPARγ. As shown in Figure 4C, following treatment with NBIF (a final concentration of 25 μM) for 72 h, both PPARα and PPARγ protein levels could be induced about 2.0-fold and 1.7-fold, respectively. Besides, to further confirmed the induction was mediated by PPARα and PPARγ, we silenced PPARα and PPARγ by siRNA in living HepG2 cells. As shown in Figures 4D–F, after knockdown of PPARα or PPARγ with their specific siRNA, the mRNA, protein expression and activity of UGT1A1 are all decreased compared to siNC group, which indicated both PPARα and PPARγ participated in the regulation of UGT1A1 expression. Collectively, these results provided solid evidence that that NBIF induced UGT1A1 *via* both activating and up-regulating PPARα and PPARγ.

### Molecular Docking and Molecular Dynamics Simulations

**FIGURE 5:**
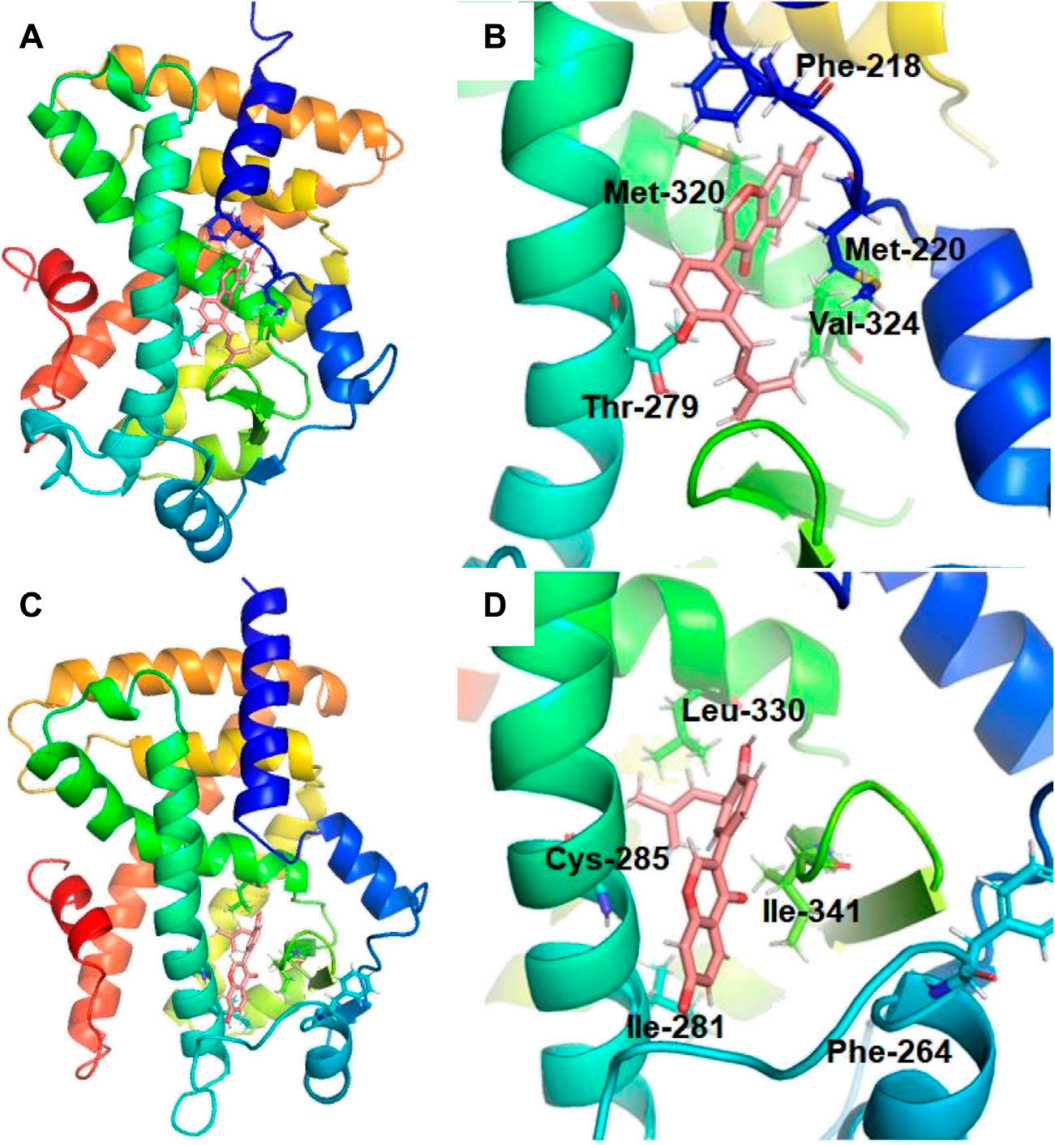
Equilibrium stereo overview (left) and the detailed view (right) of NBIF binding on various PPAR isoforms, including PPARα **(A)** and PPARγ **(B)**.

**FIGURE 6:**
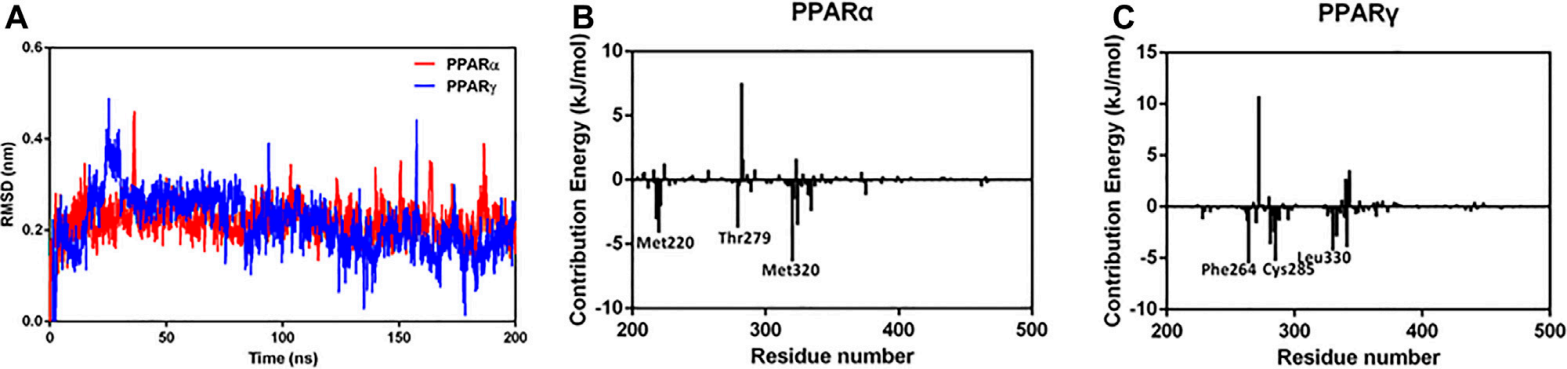
The RMSD of NIBF binding to PPARα and PPARγ during 200 ns simulations **(A)** and the binding energy contribution of individual residues in PPARα **(B)** (residue 202–468) and PPARγ**(C)** (residue 207–477).

To further explore the microscopic mechanisms of NBIF on the PPARα and PPARγ, molecular docking and molecular dynamics simulations were performed, using the ligand-binding domains (LBDs) of PPARα and PPARγ. As shown in Figure 5 and Supplementary Figures S1,S2, NBIF could bind on PPARα and PPARγ at the region between H3 and the β sheet, which was referred to as pocket II with hydrophobic interior and entrance (Zoete et al., 2007). To further investigate the protein-ligand interactions, the stability under a finite temperature, 200 ns molecular dynamics simulations of PPAR-NBIF complexes were performed. As shown in Figure 6A and Supplementary Figure S3, the Root-Mean-Square Deviations (RMSDs) values indicated stable binding for NBIF on the PPARα and PPARγ. The total binding energy and decomposition energy of each residue were also calculated using the MM/PBSA method. The binding energies were −58.814 kJ/mol and −77.127 kJ/mol for NBIF binding on PPARα and PPARγ, respectively (Table 3). As shown in Table 3 and Figures 6B,C, the hydrophobic interactions contributed predominantly to the binding of NBIF on PPAR isoforms, such as Phe218, Met220, Thr279, Met320 and Val324 of PPARα, as well as Phe264, Ile281, Cys285, Leu330 and Ile341 of PPARγ.

**TABLE 3:** The binding free energies calculated by MM-PB/SA method.

Energy (kJ/mol)^a^	PPARα	PPARγ
Δ*E*_elec_	−64.0	−69.9
Δ*E*_vdw_	−141.5	−171.6
Δ*E*_polar_	164.1	184.7
Δ*E*_nonpolar_	−17.4	−20.4
Δ*G*_bind_	−58.8	−77.1

^a^ The binding free energy of 500 snapshots retrieved from the last 100 ns equilibrium trajectories was calculated by the MM-PB/SA method. Δ*E*_elec_ referred to the energy of electrostatic interactions. Δ*E*_vdw_ referred to the energy of van der Waals interactions. Δ*E*_polar_ and Δ*E*_nonpolar_ referred to the electrostatic and non-electrostatic contributions to the solvation energies, respectively. Δ*G*_bind_ referred to the total binding free energy. The entropy contributions were ignored due to relatively small structural fluctuations.

## Discussion

Over the past few decades, the biological roles of UGT1A1 in metabolic clearance of both endogenous toxins (such as bilirubin) and therapeutic drugs have been well-established (Tukey and Strassburg, 2000a). UGT1A1 is essential for detoxification of bilirubin and play a central role in the metabolism of some important therapeutic drugs, while a common mutation in the TATA box of its promoter reduces the expression level of the enzyme and makes it more susceptible to drug-drug interactions or drug-endobiotic interactions(Rabenstein et al., 1999). However, studies the past 5–10 years have shown that mild hyperbilirubinemia is safe and actually has a health benefit. This is especially prevalent in humans with the Gilbert’s polymorphism that have mild hyperbilirubinemia and no liver dysfunction. Studies have found that humans with Gilbert’s are at reduced risk of obesity, diabetes, and cardiovascular diseases. Other studies have now shown that people with obesity and diabetes have reduced levels of plasma bilirubin. There has been a recent finding that bilirubin has beneficial effects via the PPARα nuclear receptor and functions as a hormone (Stec et al., 2016; Gordon et al., 2019; Gordon et al., 2020). These findings have shifted the scientific thinking of bilirubin and hyperbilirubinemia. Having efficient as well as safe UGT1A1 inducers for ameliorating hyperbilirubinaemia and drug-induced liver toxicity is one of the ways to overcome such medical problems. However, until now, safe and potent UGT1A1 inducers are rarely reported. Currently, there is no ideal medication can be used for the prevention and treatment of hyperbilirubinaemia or other UGT1A1 deficiency associated disorders in clinical setting. Thus, it is necessary to find more efficacious UGT1A1 inducers for both basic researches and translational applications. The purpose of this study was to find such UGT1A1 inducers from natural or semi-synthetic flavonoids, owing to that these compounds display good safety profiles. For this purpose, a series of flavonoids (including flavones and isoflavones) have been assayed for their inductive effects on the human UGT1A1. Among all tested flavonoids, neobavaisoflavone (NBIF), a natural isoflavonoid isolated from the dried fruit of *Psoralea corylifolia* L., was found to exhibit the most potent inductive effects, encouraging us to further investigate the details and underlying molecular mechanism of NBIF effect on UGT1A1 expression.

Our results demonstrated that NBIF could up-regulate human UGT1A1 in both intestinal and hepatic cell lines *via* dose- and time-dependent manners (Figure 1). The inductive effect of NBIF in HepG2 cells is more potent than in Caco-2 cells at both the transcription level and particularly protein/activity levels (Figure 2). We further explore the molecular mechanism of NBIF on UGT1A1 expression and the results suggested that it is mainly *via* activating PPARs rather than either AhR, PXR or FXR (Figure 3). It has been reported that PPARα is the major PPAR isoform in both HepG2 and Caco-2 cells, but the abundance of PPARα in HepG2 is much higher than that in Caco-2 cells (Falamic et al., 2017). Thus, the differences with UGT1A1 induction in HepG2 and Caco-2 cells can be partially attributed to the differential expression of PPARα in these two cell lines. Furthermore, these findings may suggest that NBIF is more active in up-regulation of hepatic UGT1A1 in the human body. In light of that the liver plays a predominant role in metabolic clearance of circulating bilirubin, NBIF can be developed as an injection agent to up-regulate hepatic UGT1A1 directly.

In addition to UGT1A1 induction, NBIF may also be regulating other key enzymes that participate in the metabolism drugs and endogenous compounds *via* activating and up-regulating PPARs. As a key ligand-modulated transcription factors, PPARs control the transcriptional rates of a wide range of genes that are implicated in multiple biological processes, such as lipid and glucose homeostasis, xenobiotic metabolism, inflammation, cell proliferation and differentiation. PPARα has been validated as a key regulator or controller of lipid metabolism (Brown and Plutzky, 2007; Willson et al., 2000). By contrast, PPARγ is a regulator of lipid and glucose homeostasis (Janani and Ranjitha Kumari, 2015). Additionally, PPARs also control the transcriptional rates of various genes encoding drug transporters and drug-metabolizing enzymes, such as UGTs, CYPs, GSTs, and SULTs (Wallace and Redinbo, 2013). In this study, we find that NBIF is a new PPARα/γ dual agonist, and this agent can dose-dependently activate PPARα and PPARγ (Figure 4). In addition, NBIF could also up-regulate protein expression of PPARα and PPARγ. It is easily conceivable that NBIF can up-regulate the transcription of a wide range of enzymes/proteins *via* activating and inducing PPARs, which in turn, regulate metabolic clearance of a variety of xenobiotics and endogenous substances. As a PPAR agonist and inducer, NBIF might serve as lead compound for the development of drug(s) to treat a range of human diseases such as diabetes, hyperglycemia, inflammation and various types of cancer (Mirza et al., 2019).

From the perspective of drug development, NBIF possesses good safety profiles and acceptable drug-likeness properties (Wang et al., 2015; Yang et al., 2018). Several previous studies have reported that NBIF could be rapidly absorbed into the circulation following oral administration of the crude extract of *Psoraleae fructus* in rats (Wang et al., 2015; Yang et al., 2018). Recently, Yang’s group investigated the pharmacokinetics behaviors of major constituents of *Psoraleae fructus* in Sprague-Dawley rats after oral administration, and reported that the plasma half-life of NBIF was 8.88 ± 5.15 h (Yang et al., 2018). In the present study, our results showed that NBIF could efficiently activate PPARs in living cells, implying that it was cell-permeable and could bind to intracellular receptors. Additionally, *in vitro* metabolic stability assays showed that NBIF possesses acceptable metabolic stability in human liver preparations (Supplementary Figure S5). These data suggested that NBIF could serve as a promising lead compound for the development of the novel UGT1A1 inducer, *via* simultaneously improving the inductive potency and drug-likeness properties. From the viewpoint of medicinal chemistry, NBIF bears several phenolic groups which can be easily modified to generate a series of esters or other derivatives. In future, it will be necessary to semi-synthesize a range of structurally diverse NBIF derivatives for studying their structure-activity relationships as UGT1A1 inducer.

## Conclusion

In summary, this study reported an efficacious natural occurring UGT1A1 inducer and revealed its inductive mechanism. Following screening of the UGT1A1 inductive effects of 40 flavonoids, NBIF was found with the most potent inductive effect on UGT1A1 and its potency was more potent than the known UGT1A1 inducer chrysin at the transcription level. NBIF could induce UGT1A1 in both Caco-2 cells and HepG2 cells, while its inductive effects on UGT1A1 were further confirmed by western blotting and NHPN *O*-glucuronidation activity assays. Further investigations demonstrated that NBIF up-regulated human UGT1A1 mainly *via* activating and up-regulating PPARs. Molecular simulations suggested that NBIF could stably bind on PPARα and PPARγ at the region between H3 and the β sheet *via* hydrophobic interactions. Collectively, our findings suggested that NBIF was a naturally occurring UGT1A1 inducer, which induced human UGT1A1 *via* activating and up-regulating PPARα and PPARγ. All these findings suggest that NBIF can be used as a promising lead compound for the development of more efficient UGT1A1 inducers.

## Data Availability

The original contributions presented in the study are included in the article/Supplementary Material, further inquiries can be directed to the corresponding authors.
