# Supplementary material for: Neobavaisoflavone Induces Bilirubin Metabolizing Enzyme UGT1A1 via PPARα and PPARγ
Source: Front Pharmacol. 2021 Feb 8;11:628314. doi: 10.3389/fphar.2020.628314 (PMC7897654; doi:10.3389/fphar.2020.628314)
Supplement: Supplementary file 1 [file table1.docx]

**Supplementary Materials**

*for*

**Neobavaisoflavone induces bilirubin metabolizing enzyme UGT1A1 via PPARα and PPARγ**

Ya-Di Zhu ^a,1^, Xiao-Qing Guan ^b,1^, Jing Chen ^b^, Sheng Peng ^a^, Moshe Finel ^c^, Ying-Yuan Zhao ^d^, Rui-Min Wang ^d^, Hui-Chang Bi ^d^, Ming Lei ^a,*^, Dan-Dan Wang ^b,*^, Guang-Bo Ge ^b,*^

^a^ The seventh affiliated people's hospital of Shanghai university of traditional Chinese medicine, Shanghai, 200137, China.

^b^ Institute of Interdisciplinary Integrative Medicine Research, Shanghai University of Traditional Chinese Medicine, Shanghai, 201203, China.

^c^ Division of Pharmaceutical Chemistry and Technology, Faculty of Pharmacy, University of Helsinki, 00014, Finland.

^d^ School of Pharmaceutical Sciences, Sun Yat-sen University, Guangzhou, 510006, China.

*Corresponding authors.

E-mail address: geguangbo@dicp.ac.cn (G.-B. Ge) [& wangdandan801@126.com (D.-D](mailto:&%20wangdandan801@126.com(D.-D). Wang) & leiming6891@163.com (M. Lei)

^1^ These authors contributed equally to this work.

This file contains one supplementary scheme, one supplementary table and five supplementary figures.

**Scheme 1.** Structure of NHPN and its application for sensing UGT1A1 activity.

**Table S1**.Sequences for UGT1A1 and GAPDH primer.

| Gene | Sequences (5’-3’) | | |
| --- | --- | --- | --- |
| h*Ugt1a1* | CCTTTGCCTCAGAATTCCTTC | | |
|  | ATTGATCCCAAAGAGAAAACCAC | | |
| hGAPDH | TCCATGACAACTTTGGTATCG | | |
|  | TGTAGCCAAATTCGTTGTCA | | |
| siPPARα | GUAGCGUAUGGAAAUGGGUUUTT |  |  |
|  | AAACCCAUUUCCAUACGCUACTT |  |  |
| siPPARβ/δ | GUGUGGAAGCAGUUGGUGAAUTT |  |  |
|  | AUUCACCAACUGCUUCCACACTT |  |  |
| siPPARγ | GACAACAGACAAAUCACCAUUTT |  |  |
|  | AAUGGUGAUUUGUCUGUUGUCTT |  |  |


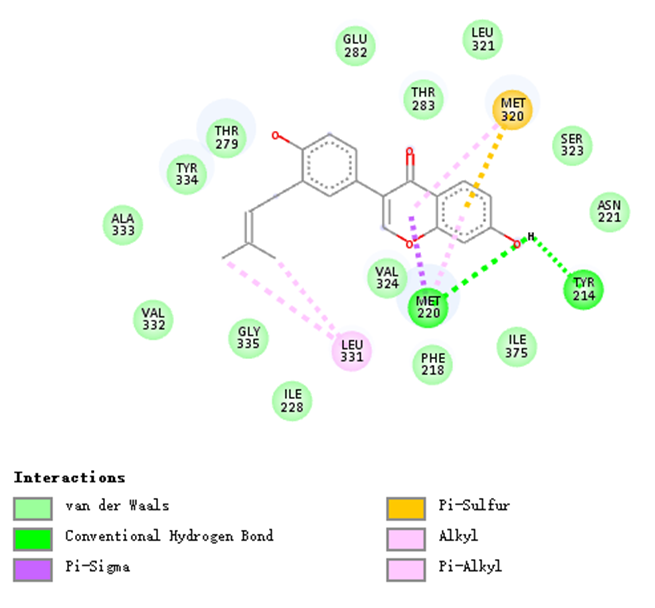


**Figure S1.** 2D representation of the interactions between NBIF and the residuals of PPARα.


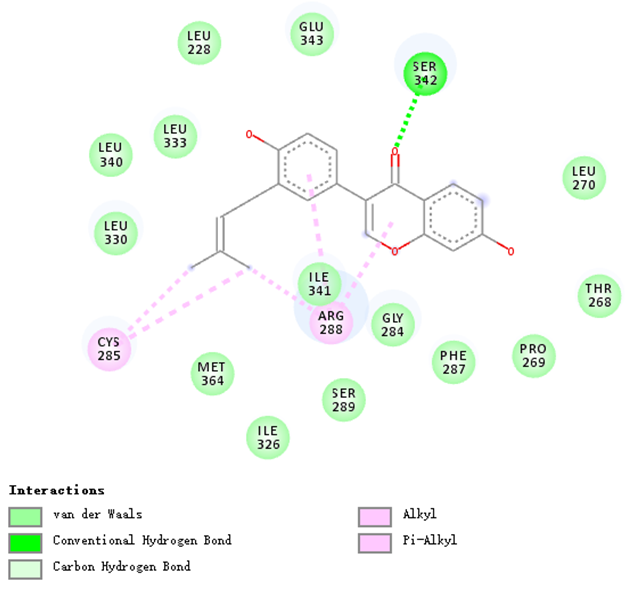


**Figure S2.** 2D representation of the interactions between NBIF and the residuals of PPARγ.

**
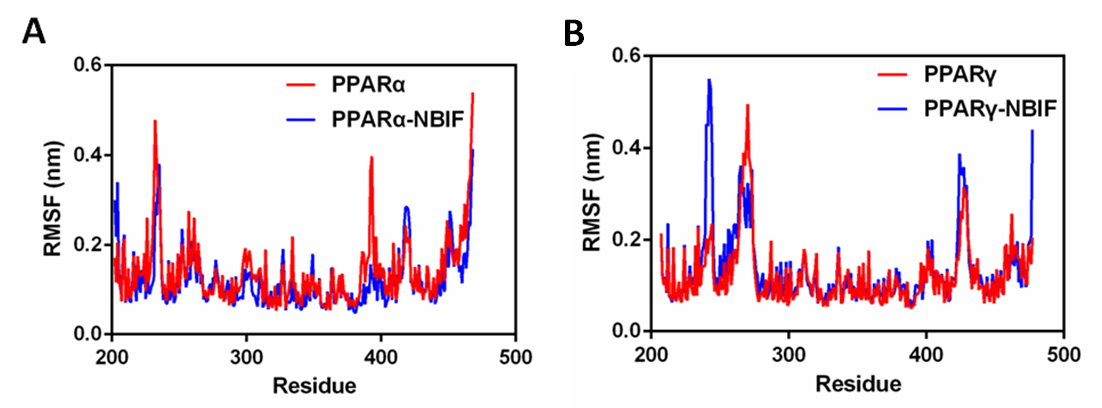
Figure S3.** The RMSF of NIBF binding to PPARα and PPARγ during 200 ns simulations.

**Figure S4.** Cytotoxicity of neobavaisoflavone (NBIF) against Caco-2 and HepG2 cells.


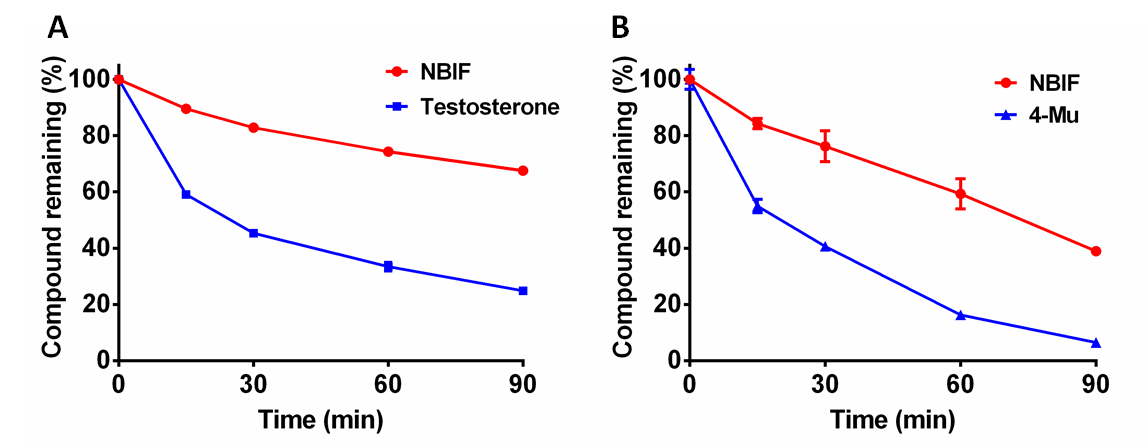


**Figure S5.** The half-life of NBIF in both phase I (A) and phase II metabolic systems (B). HLM is used as the enzyme source, while testosterone is used as the positive substrate for phase I metabolism and 4-methylumbelliferone (4-Mu) is used as the positive substrate for phase II metabolism.
